# A review of luteinising hormone and human chorionic gonadotropin when used in assisted reproductive technology

**DOI:** 10.1186/1477-7827-12-95

**Published:** 2014-10-03

**Authors:** Diego Ezcurra, Peter Humaidan

**Affiliations:** EMD/Merck Serono, One Technology Place, Rockland, MA 02370 USA; Skive Regional Hospital and Faculty of Health, Aarhus University and Odense University, Resenvej 25, Skive, 7800 Denmark

**Keywords:** Luteinising hormone, Human chorionic gonadotropin, Human menopausal gonadotropin, Assisted reproductive technology, Implantation

## Abstract

Gonadotropins extracted from the urine of post-menopausal women have traditionally been used to stimulate folliculogenesis in the treatment of infertility and in assisted reproductive technology (ART). Products, such as human menopausal gonadotropin (hMG), consist not only of a mixture of the hormones, follicle-stimulating hormone (FSH), luteinising hormone (LH) and human chorionic gonadotropin (hCG), but also other biologically active contaminants, such as growth factors, binding proteins and prion proteins. The actual amount of molecular LH in hMG preparations varies considerably due to the purification process, thus hCG, mimicking LH action, is added to standardise the product. However, unlike LH, hCG plays a different role during the natural human menstrual cycle. It is secreted by the embryo and placenta, and its main role is to support implantation and pregnancy. More recently, recombinant gonadotropins (r-hFSH and r-hLH) have become available for ART therapies. Recombinant LH contains only LH molecules. In the field of reproduction there has been controversy in recent years over whether r-hLH or hCG should be used for ART. This review examines the existing evidence for molecular and functional differences between LH and hCG and assesses the clinical implications of hCG-supplemented urinary therapy compared with recombinant therapies used for ART.

## Background

**Figure 1:**
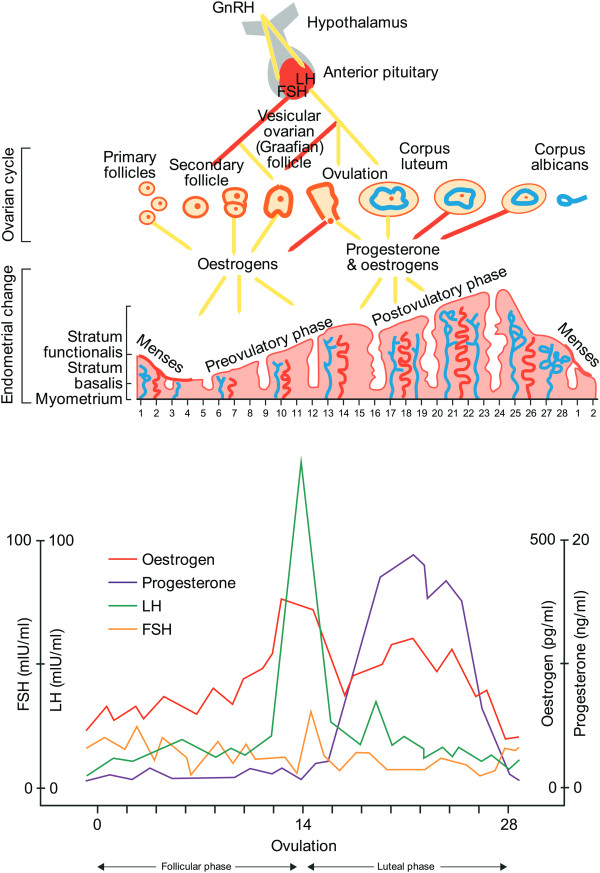
**Hormones act in concert to regulate normal ovarian function.**

The human gonadotropins, luteinising hormone (LH), follicle-stimulating hormone (FSH) and human chorionic gonadotropin (hCG), are complex heterodimeric glycoprotein hormones that each play pivotal, though differing, roles in the female reproductive system. Normal ovarian function depends on the concerted action of FSH and LH, both of which are produced in the anterior pituitary. According to the two-cell two-gonadotropin theory, these hormones — together with local steroidal and non-steroidal factors — stimulate follicular growth and maturation, ovulation, and the development of the corpus luteum [1] (Figure 1).

In contrast, hCG only becomes important once there is an embryo, such that hCG produced by the developing embryo takes over from LH in the upregulation of the corpus luteum progesterone production. Between 3 and 4 weeks after implantation, the placenta becomes capable of progesterone production independently of hCG and the role of hCG then changes to one focused on the promotion and maintenance of the maternal blood supply to the developing foetus. This is achieved through hCG binding to the LH/hCG receptors on the uterine spiral arteries and the subsequent promotion of angiogenesis. Additionally, hCG is involved in the differentiation of placental cells, prevention of rejection of foetal-placental tissue and the promotion of uterus growth in line with foetal growth [2].

While LH and FSH are each single molecular entities, hCG exists in a number of different molecular forms [2, 3]. In addition to regular hCG, variants include sulphated pituitary hCG that is present at low levels during the menstrual cycle and in post-menopausal women; a hyperglycosylated form that promotes growth and invasiveness of cytotrophoblast cells during embryo implantation and in malignancy; and also as a free hCG β-subunit, an autocrine factor produced in many malignancies. These functional and molecular differences between LH and hCG suggest that there may be differences in the clinical efficacy of the two gonadotropins.

Historically in the treatment of infertility and in assisted reproductive technology (ART), urinary human menopausal gonadotropin (hMG) from post-menopausal women has been used to stimulate folliculogenesis. Over the years, a number of working hypotheses have developed concerning the advantages and disadvantages of the various exogenously added gonadotropin supplements used in ART, particularly with respect to hMG. Although urinary-derived hMG contains both FSH and LH, the LH content is highly variable and hMG is supplemented with urinary hCG, which is intended to mimic the action of LH and to standardise the product.

Technological advances have led to the ability to produce recombinant forms of human FSH (r-hFSH) and LH (r-hLH). These are now available as potential alternatives to hMG and may be more suitable in those subpopulations of patients requiring the addition of LH in their stimulation protocols. This is of particular relevance given the increasing recognition of the need for individualised controlled ovarian stimulation (iCOS) to maximise the benefit for patients.

The aim of this review is to present evidence from both laboratory-based, *in vitro* and clinical studies in order to identify any differences between LH and hCG at the molecular and functional levels and to examine the implications that these differences may have on clinical outcomes.

## Review

### The nature of hCG-containing hMG products

#### The origin of hCG in hMG

A commonly held belief is that all the hCG contained in high-purity hMG (HP-hMG) comes naturally from the urine of post-menopausal women and that no exogenous hCG is added to these HP-hMG treatments. However, there is evidence to show that as the purity of a hMG preparation is increased, more LH molecules are preferentially lost [4] and more exogenous hCG needs to be added to return to the required FSH:LH ratio of 1:1 in the original product [5]. In a study of commercially available urinary hMG products [4], the older less purified products (e.g. Pergonal) contained more endogenous LH and less exogenous hCG (Table 1). In contrast, the majority of LH bioactivity in HP-hMG was provided by hCG supplementation [4]. In earlier analyses of this same HP-hMG, Giudice et al. [6] found the hCG content to be 10-fold higher than that of LH, while van de Weijer et al. [5] reported three times as much immunoreactive hCG to be present as LH (Table 1). The authors of the latter study concluded that 95% of the LH bioactivity in the HP-hMG was due to the presence of hCG. Although post-menopausal women release core β-fragments of hCG in urine, van de Weijer et al. [5] concluded that the relatively high amount of hCG in the HP-hMG can only be explained by assuming the addition of hCG from external sources, a well-established practice in the production of hMG for standardisation purposes. This means that when hCG is added to hMG to increase its LH bioactivity, the variability in “real LH” content and the significantly longer half-life of hCG make it more difficult to control the administered product. In contrast, pure LH, sources of which include r-hLH alone or in combination with r-hFSH, allow for more precise dosing and physiological action to control follicular development.

**Table 1:** **LH and hCG content (immunoreactivity) of different urinary hMG preparations**

Product	LH IU/vial (SD)	hCG IU/vial (SD)	LH/hCG ratio	Study
*Pergonal*	13.49 (3.6)	3.39 (1.7)	3.98	Wolfenson et al. [4]
*Humegon*	5.77 (1.0)	6.86 (1.8)	0.84	Wolfenson et al. [4]
*Menopur*	0.29 (5.2)	9.61 (2.3)	0.03	Wolfenson et al. [4]
*Menopur*	0.48 (1.7)	9.05 (3.3)	0.05	Wolfenson et al. [4]
*Menopur*	0.39 (3.1)	11.06 (1.8)	0.04	Wolfenson et al. [4]
*Menopur*	0.85 (0.18)	11.3 (1.0)	0.08	Giudice et al. [6]
*Menopur*	3 (range 2.7–5.3)	10 (range 9.9–11.2)	0.03	Van de Weijer et al. [5]

*hCG* human chorionic gonadotropin; *IU* international units; *LH* luteinising hormone; *hMG* human menopausal gonadotropin.

#### The purity of hMG

Eight purification steps, including hormone absorption and elution, anion and cation exchange, and hydrophobic chromatography are used in the preparation of HP-hMG. Despite this, urinary hMG products not only contain hCG, but also relatively high percentages of other protein impurities and these impurities vary between batches [5, 6]. Van de Weijer et al. [5] used reverse-phase high-performance liquid chromatography (HPLC) analysis and 2D gel electrophoresis to identify at least 30% protein impurities in an hMG preparation, including leucocyte elastase inhibitor, protein C inhibitor, and zinc-α_2_-glycoprotein.

Other studies have also found contaminant proteins such as growth factors, glycoproteins, binding proteins, transferrin, and immunoglobulins that are not reported to induce follicular development and which may influence the efficacy and possible safety of hMG products [7, 8]. Some of the contaminants identified were highlighted as being biologically active (e.g. epidermal growth factor [EGF], tumour necrosis factor binding protein-1 and Tamm–Horsfall glycoprotein). Their presence exposes patients to possible adverse effects [7]. For example EGF is a potent mitogenic factor, not normally present in the follicular phase of the ovulation cycle that induces stromal and epithelial cell proliferation and differentiation. While *in vitro* studies have reported that EGF increases cumulus cell numbers and oocyte maturation [9], the exogenous EGF in hMG could interfere with the normal process of cell proliferation and differentiation in endometrial cells by synergising with other growth factors and replacing the actions of oestradiol. It might also compete with other EGF-like factors reported to play roles in oocyte development and ovulation [10].

Another potential contaminant of hMG, insulin-like growth factor (IGF)-binding protein-7 regulates cellular proliferation, adhesion, and angiogenesis, and may suppress oestrogen production by granulosa cells [11]. None of these contaminants appear to confer any proven advantage in terms of clinical outcome and, moreover, the concentration and type of impurities varies considerably between batches [6].

Of particular interest are two recent proteomic analyses of the composition of urinary-derived hMG, hCG and HP-hMG that detected the presence of prion proteins [12, 13]. Van Dorsselaer et al. [13] identified prion protein as a ‘major contaminant’ of hMG preparations, while prion proteins were not detected in recombinant products. Prions are a mis-folded isoform of a normal cellular protein found in the brain (PrPc), and are associated with transmissible spongiform encephalopathies (TSEs). When a prion comes into contact with another native version of this protein, it induces the native protein to adopt the mis-folded shape. As the body is unable to recognise and break down the abnormally folded protein, prions accumulate in the central nervous system, interfering with normal brain function. Conversion of PrPc into the abnormal form can occur spontaneously or following infection. Abnormal prions have been identified including PrPsc, which is the protein associated with scrapie, and PrPres, the protein resistant to enzyme degradation found in patients with Cruetzfeldt-Jacob disease (CJD) [14].

Although the studies by Kuwabara et al. [12] and Van Dorsselaer et al. [13] showed that prion proteins can be detected in urinary-derived fertility preparations including hMG, there is no strong evidence to support the suggestion that vCJD (or sporadic CJD) has been acquired through receiving urinary gonadotropins [15]. The risk of TSE from urinary products has even been described as unproven or theoretical [16, 17]. However, as described by Van Dorsselar in the absence of evidence for no risk of urinary TSE transmission, the precautionary principle should be considered for urinary-derived preparations [13]. In the end when treating patients for ART we intended to inject only gonadotropins like FSH, LH and hCG but not contaminants, to act at the level of the ovarian axis, develop follicles and mature oocytes that will be retrieved for later fertilization and the production of embryos for transfer.

### Comparison of LH and hCG at the molecular level

While hCG can mimic the bioactivity of LH, there are differences between LH and hCG at the molecular level. However, both are members of the cystine-knot growth factor families of highly glycosylated, non-covalently linked α- and β-subunits that exhibit the properties of cytokines and chemokines [18]. LH originates from the pituitary gland and is a heterodimeric glycoprotein with a molecular mass of 28 kDa comprising alpha (92 amino acids) and beta (120 amino acids) subunits. Whereas hCG, also a heterodimer, is composed of 244 amino acids with a molecular mass of 36.7 kDa.

Different cell types produce different forms of hCG. Regular hCG is produced by the placental syncitiotrophoblasts and hyperglycosylated hCG is produced by stem cytotrophoblastic cells, while sulphated hCG is made in small amounts by the pituitary and the free β-subunit is produced by non-trophoblastic malignancies [2, 19]. The alpha subunit of hCG comprises 92 amino acids and is almost identical to that of LH [18]. Although the α-subunits of LH and hCG show a high degree of similarity, the β-subunit is unique to each hormone. As well as containing an extra 24 amino acids, the β-chain of hCG possesses additional glycosylation sites, eight for hCG compared with three for LH. The extra glycosylation sites give hCG a longer half-life (terminal half-life via the subcutaneous route of 32–33 hours for recombinant hCG vs 21–24 hours for r-hLH [20, 21]). It is the β-subunit of the hormone that confers its specificity and particular physiological activity [22].

### Comparison of the functional properties of LH and hCG

#### Roles in follicular development

Some proponents believe that hCG is ideal for follicular development because its long half-life provides a more sustained LH stimulation. Recombinant LH, in contrast, has a shorter half-life and, according to some authors, requires multiple daily injections to sustain follicle development [2]. In addition, hCG has a higher binding affinity for the receptor and is more potent [23]. These observations have led to the assumption that the more hMG (containing hCG) is added during stimulation, and the earlier that this occurs (i.e. at day 1), the more favourable the outcome will be.

Although binding to the same receptor, LH and hCG play different physiological roles within ovulation and pregnancy. These differing roles are reflected by the timing of each hormone’s appearance in the ovulation cycle [2]. Initially, FSH acts on the granulosa cells [24] of the ovary to stimulate follicular development and the aromatisation of androgens for the production of oestradiol while LH interacts with the theca cells for androgen production, the raw material for oestrogen synthesis. At a follicle size of 8–12 mm, under the effect of FSH the granulosa cells express LH receptors, enabling LH to have, along with FSH, a major role in follicular growth and oocyte maturation. The surge of LH at mid-cycle induces ovulation, resumption of meiosis in the oocyte [25], formation of the corpus luteum, luteinisation of the theca and granulosa cells, and early progesterone synthesis [26]. Unlike LH, hyperglycosylated hCG is produced by the trophoblastic cells of the early embryo (days 4–6) to stimulate the corpus luteum to progesterone production and initiation of the implantation process. After 3–4 weeks the placenta starts to produce progesterone [2]. The placenta then takes over hCG production and hCG levels peak after 10 weeks of gestation.

LH and hCG possess significantly different *in vitro* biopotencies, despite binding to the same receptor, as shown in a study using COS-7 cells expressing the LH/hCG receptor [23]. In this study, the effective dose at 50% (ED_50_) of the maximal cyclic adenosine monophosphate (cAMP) response was approximately 5-fold greater with hCG than with equimolar concentrations of LH. In addition, use of equipotent ED_50_ concentrations of LH and hCG showed that the cAMP response to LH reached a plateau after 10 minutes vs 1 hour for hCG. Continuous exposure to LH and hCG for 12 hours revealed repetitive and pulsatile increases in cAMP activation every 3–4 hours and significantly higher levels of stimulation by hCG compared with LH [23].

**Figure 2:**
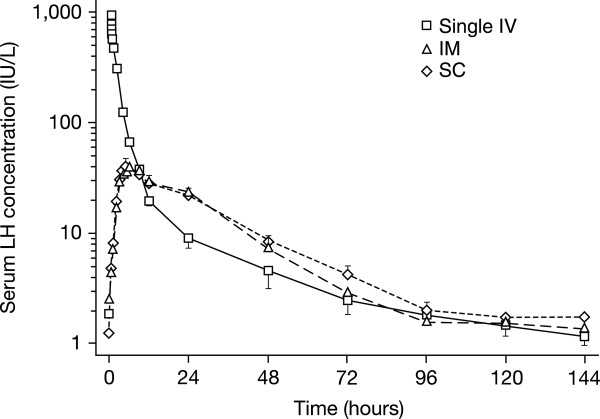
**LH immunoassay concentrations over time after three routes of administration.** Log-linear plot of LH immunoassay concentrations over time after Single IV (solid line), IM (long dashed line), and SC short dashed line administration of 10,000 IU of r-hLH (mean ± 1 SEM, 12 subjects). Reprinted from Fertil Steril, 69, le Cotonnec JY, Porchet HC, Beltrami V, Munafo A, Clinical pharmacology of recombinant human luteinizing hormone: Part II. Bioavailability of recombinant human luteinizing hormone assessed with an immunoassay and an in vitro bioassay, pages 195–200, Copyright 1998, with permission from Elsevier [20].

In addition to these pharmacodynamic differences, the pharmacokinetics of LH and hCG are also quite different. After intravenous (IV) administration, the pharmacokinetics of r-hLH follow a two-compartment model, while after subcutaneous (SC) administration (Figure 2), the pharmacokinetics of r-hLH can be described by using a one-compartment model with zero-order absorption and a lag time [20]. Following IV administration, r-hLH undergoes a rapid distribution phase with an initial half-life (distribution half-life) of approximately 1–1.3 hours (as assessed by immunoassay and *in vitro* bioassay), and a slower elimination phase with a terminal half-life (elimination half-life) of around 10–19 hours [20]. After SC administration, the terminal half-life was approximately 21–24 hours as assessed by immunoassay and *in vitro* bioassay. With an effective half-life of approximately 1 day, r-hLH is suitable for once-daily SC injections resulting in only a modest accumulation of 1.6 IU/L of LH with repeated daily administration of 150 IU of LH [20]. In contrast, the terminal half-life of hCG is 32–34 hours [21], leading to the possibility of drug accumulation. Indeed, patients treated with 50 IU/day of hCG showed measurable and increasing levels of hCG over the course of a treatment cycle to 16.2 ± 3.2 IU/L of hCG, which is equivalent to 113.4 IU/L of LH activity [27]. The accumulation of hCG over the ovulation cycle can lead to increased interaction at the LH/hCG receptor level. This is of importance since a threshold level of 1.2 IU/L of LH is required for optimal follicular development [28, 29]. There is a ‘ceiling’ LH level (5 IU/L) above which granulosa cell proliferation is suppressed and atresia (of the non-dominant follicles) and premature luteinisation (of the pre-ovulatory follicle) occurs [30, 31].

High levels of LH also lead to desensitisation and down regulation of the LH receptors [32, 33]. This occurs transiently under physiological conditions (the pre-ovulatory LH surge) or in response to pharmacological doses of hCG [34]. Down regulation of the LH/hCG receptor is mediated by the accelerated degradation of *LH/hCG receptor* mRNA caused by the LH receptor mRNA binding protein mevalonate kinase [33]. This has been confirmed in gene expression studies where there was consistently lower expression of the LH/hCG receptor gene in granulosa cells from women undergoing controlled ovarian hyperstimulation who were treated with hMG compared with those treated with r-hFSH [32]. Thus, the high levels of hCG contained in hMG, together with its longer half-life, may lead to an undesirable accumulation of LH-like bioactivity with possible premature luteinisation and reduced fertilisation rates [35] or to a reduced response as a result of LH/hCG receptor desensitisation.

A summary of the molecular and functional differences between LH and hCG is shown in Table 2.

**Table 2:** **Differences between LH and hCG**

	LH	hCG
Secreted by	Pituitary	Embryo and placenta
Physiological role	Support follicle development (14 days)	Support implantation and pregnancy (282 days)
Binding affinity	Lower	Higher (2x)
Half-life	Shorter (23 h sc)	Longer (32–33 h sc)
Accumulation	Slight	Significant and down regulation of LH receptor
Stimulation of LH receptor	Physiological	Pharmacological, leading to LH receptor down regulation
Equivalency	6–8 IU of LH	1 IU of hCG
Purity	99%	99% purity for r-hCG and 70% in HP-hMG (39 identified contaminants)
Sources	r-hLH	r-hCG, urinary hCG or hMG
Induction of steroid (testosterone, oestradiol and progesterone) production	Higher LH = higher steroid production	Higher hCG = higher steroid production
Filling system	Filled by mass	Filled by Mass for r-hCG and Filled by IU for hCG/hMG
Gene activation	Differential unexplained	Differential unexplained
Cytokine production	Differential unexplained	Differential unexplained
Embryo quality production	Not objectively proven	Not objectively proven

*h* hours; *hCG* human chorionic gonadotropin; *IU* international units; *LH* luteinising hormone; *sc* subcutaneous.

### The production of progesterone

The majority of circulating progesterone is produced in the intrafollicular compartment by the granulosa cells and the main driver of progesterone production is increased LH/hCG receptor activity. There is a general assumption that the hCG present in hMG provides LH-like activity, supporting the conversion of intrafollicular progesterone to estradiol. It is thought that this process cannot happen when using only r-hFSH, due to the lack of an LH component. However, several studies have shown that ART is successful with the administration of r-hFSH only, possibly due to the persistence of low levels of endogenous LH in the women treated despite the use of GnRH analogues [36–40].

Conversion of intrafollicular progesterone to oestradiol at the time of triggering final follicle maturation enables the endometrium to be more receptive to embryo implantation. This suggests that the conversion of progesterone to oestradiol, due to the effect of the hCG contained in hMG, may lead to better outcomes than r-hFSH alone. This theory originated from an underpowered post-hoc analysis of a subpopulation by the MERIT study, which found that higher serum hCG levels correlated with lower progesterone and higher pregnancy rates [41]. However, the enzymes cytochrome 17a-hydroxylase-C17,20 lyase (P450-17α) (converts free progesterone to oestradiol) and 3-β-hydroxysteroid dehydrogenase [3βHSD] (converts pregnenolone to progesterone) are localised to the ovarian thecal/interstitial cells and do not exist in the follicular compartment [42]. This finding suggests that progesterone cannot be converted to oestradiol in the follicular compartment. Further, in a comparative study of the use of urinary hMG with r-hFSH, Wolfenson et al. [4] found that progesterone levels produced in an *in vitro* follicle bioassay were 2–3 times higher with hMG than with either urinary FSH or r-hFSH.

### Association between progesterone levels and clinical outcomes

In contrast to the MERIT study, a subsequent retrospective study by Andersen et al. [43] reported a positive correlation between progesterone levels and the number of follicles and oocytes. Possible reasons for the discrepancy between these results and those of the MERIT study include a higher starting dose of FSH in the MERIT study [41, 43].

A more recent large multicentre randomised controlled trial of hMG vs r-FSH reported a significant decrease in pregnancy rate with increased progesterone level [44]. In this study, in the r-FSH group, women with progesterone levels >4 nmol/L had a significantly lower pregnancy rate compared with those women with progesterone levels ≤4 nmol/L, while in the hMG group, pregnancy rates were similar in women with progesterone levels ≤4 nmol/L or >4 nmol/L [44]. Other studies have shown that a higher progesterone/oestradiol ratio is not associated with lower pregnancy rate and, in particular, embryo implantation is favoured, due to lower uterine contractility [45, 46]. A further study of r-hFSH/r-hLH vs HP-hMG and urinary FSH reported that serum and intra-follicular progesterone levels and pregnancy rates were not significantly different between groups [47].

A randomised, controlled trial by Thuesen et al. [48], examined serum progesterone in women treated with increasing doses of hCG (0–50–100–150 IU) in combination with 150 IU of r-hFSH. In this study, supplementation with increasing doses of hCG from the first day exponentially increased pre-ovulatory progesterone and hydroxy-progesterone [48]. Moreover, in response to a Letter to the Editor, the authors concluded that the key importance of their study was to show that in the hCG dose range of 0–150 IU/day, supplementation with hCG did not seem to reduce but rather to increase late follicular phase progesterone levels [49]. Pregnancy rates were not reported in this study.

Overall while these studies demonstrate that serum progesterone increases with LH or hCG use, threshold level and timing of progesterone increase and it's clinical implications of this increase remain unclear.

### Intrafollicular cumulus gene expression and cytokine profiling

One study has concluded that the hCG contained within hMG supports the production of a different profile of cytokines and different gene expression in the cumulus and endometrial cells, generating a better environment to produce healthier oocytes compared with r-hLH [50]. The upregulated cytokines are known promoters of implantation and angiogenesis and are anti-apoptotic.

However, hCG, with its long half-life and greater receptor affinity, is likely to exceed the LH ‘ceiling’, a situation causing atresia of non-dominant follicles and LH/hCG receptor down regulation. In a comparison of gene expression studies in women undergoing controlled ovarian stimulation (COS) with either r-hFSH or hMG, there was a consistently lower expression level of the LH/hCG receptor gene in granulosa cells from the hMG treatment group compared with the r-hFSH group. Several genes involved in the biosynthesis of cholesterol and steroids were also differently regulated and showed reduced expression in the granulosa cells of the hMG-treated group [32]. This group of subjects also showed increased expression of the *S100 calcium-*binding p gene encoding an anti-apoptotic protein.

Another gene expression study of cumulus cells from patients treated with HP-hMG or r-hFSH also reported significant differences in gene expression involved in ovulation, fertilisation, EGF signalling and embryonic development depending upon the treatment [51]. Patients treated with rFSH had increased *SPROUGHTY4* but lower *SDC4* levels when compared with hMG-treated patients. In the same study, the expression of oocyte maturity (*VCAN*), progesterone and embryo development on day 3 (*GREM1*) and *RPS6KA2* was related to factors such as age in the rFSH treated patients but not in the hMG treated patients. The authors concluded that the presence of LH bioactivity in the hMG preparation might have led to a ‘damping effect on LH responsive genes’ [51].

### Production of high-quality embryos

It has been suggested that due to the hCG content, hMG produces fewer oocytes, however, they are of better quality than those stimulated with r-hFSH alone or the addition of r-hLH. Day 6 hCG concentration has been shown to predict the frequency of top-quality embryos, ongoing pregnancy and live birth rates [50]. However, single point morphological assessments of embryo quality — such as cell number, degree of fragmentation and symmetry — are subjective and imprecise and while these assessments can provide certain clues about quality, they are not able to objectively assess the physiological state, viability or implantation potential of an embryo. There is no standardised embryo grading system employed to date, and although there are reports describing subjective embryo selection methodologies which result in high implantation rates [41, 52, 53], these are not reproducible from one laboratory to another. Indeed morphological grading can vary dramatically in the course of a few hours, as shown by time-lapse observations of single embryos, and can be misleading with respect to categorising the stage of development reached [54, 55]. Therefore, objective technologies are required to identify high-quality embryos that will increase the chance of producing successful pregnancies and live births.

Recent studies using gene expression profiling have made advances in the identification of potential biomarkers to identify oocytes that should produce top-quality embryos. Paracrine activity between the oocyte and the cumulus cells is essential to ensure the competence of the developing oocyte and subsequent embryonic development [56]. Thus, initial studies focused on gene expression profiling of the cumulus cells at specific phases of oocyte development [51, 57–61] or at varying stages of oocyte maturation [62] using a variety of genes as potential biomarkers of embryo development. In one such study *HAS2, PTGS2 and GREM1* expression by cumulus cells correlated with the number of higher grade embryo [60]. Increased expression of the *Pentraxin 3* gene has also been identified as being of potential relevance for identifying good quality embryos [63]. Later studies have provided data on the gene expression of oocytes themselves. One study showed that genes related to cell cycle regulation, chromosome alignment, sister chromatid separation, oxidative stress and ubiquitination were different between younger and older oocytes [64]. A further review of human embryo gene expression profiles revealed that components of the Wnt and transforming growth factor-β signalling pathways were linked to oocyte maturation and embryo development [65].

Genomic markers of oocyte viability appear to be better with r-hFSH and r-hLH compared with hMG. r-hLH supplementation in a long GnRH agonist protocol has been shown to reduce granulosa cell apoptosis as measured by DNA fragmentation rate and caspase-3 activity (p <0.01) compared with either r-hFSH or urinary FSH/urinary hCG, indicating the production of higher quality oocytes [66]. In a recent study on the use of r-hFSH vs hMG, Wathlet et al. [67] concluded that gene expression profiling could be used to identify top-quality oocytes. *SDC4* and *TRPM7* gene expression at days 3 and 5 were identified as being the most predictive for good embryos in the FSH-treated women while *VCAN* gene expression had a negative predictive value. In addition, a significant upregulation of PTGS2 and a non-significant trend for increased ITPKA was observed in the FSH group [68].

### Clinical effects of LH supplementation

As discussed in the previous section, it has been suggested that due to the hCG content of hMG, fewer but better quality oocytes are produced, leading to high quality embryos, better endometrial receptivity and higher implantation leading to improved live birth rates [50]. However, to date no studies of sufficient power have been performed to compare the effects of LH and hCG on pregnancy and take-home baby rates. The impact of r-hLH administration in oocyte donors on clinical pregnancy rates in recipients was reported in a study by Acevedo et al. [69]. Oocyte and embryo quality, and fertilisation and implantation rates were all significantly higher in recipients of embryos from donors receiving r-hFSH and r-hLH plus gonadotropin-releasing hormone (GnRH) antagonist compared with r-hFSH and GnRH antagonist alone. The clinical pregnancy rate was also higher with r-hLH supplementation (51% vs 30%), although this difference was not statistically significant. A subsequent comparison of the effect of r-hLH and r-hFSH reported a trend for increased implantation with the use of r-hLH compared with r-hFSH [66]. A lower apoptosis rate was also detected in the r-hLH-treated patients. Subsequent studies supported the use of r-hLH to improve the outcome of ART [67, 70]. The addition of r-hLH to r-hFSH resulted in increased implantations in women under 35 (from 14.2% to 23.2%, p = 0.05) and, although not significant, an increase in live births in this age group from 24.4% to 28.9% [67]. Paterson et al. [70] further confirmed the importance of LH supplementation for increased pregnancy and birth rates. This large, retrospective study of 1565 *in vitro* fertilisation (IVF) or intra-cytoplasmic sperm injection (ICSI) cycles found the rates of pregnancy (61% and 54%, p = 0.006) and live births (49% and 42%, p = 0.01) for the use of a combination of r-hLH and r-hFSH, and r-hFSH alone, respectively. Improvements in fertilisation and implantation were also observed.

Some previous studies investigating the effect of exogenous r-hLH had concluded that there was no benefit in terms of increased pregnancy rates [71–73]. However, it is, possible that the relatively low numbers in the study by Kolibianakis et al. [71] were insufficient to reach clinical significance. While Mochtar et al. [72] reported no significant difference with r-hLH supplementation; these authors did find that there was a trend for r-hLH to have a beneficial effect especially a reduced rate of early pregnancy loss in poor-responders. GnRH agonist down-regulated women with baseline LH levels <0.5 mIU/ml undergoing ART, treated with r-hLH, in addition to r-hFSH, had a lower intrafollicular concentration of vascular endothelial growth factor (VEGF), a marker of apoptotic potential), elevated oestradiol levels, and increased fertilisation and pregnancy rates compared with women receiving r-hFSH alone [74]. The addition of r-hLH also increased the production of follicular adiponectin [75], which may enhance follicular insulin sensitivity, potentially leading to lower insulin levels and decreased androgens, resulting in a better follicular environment. In contrast to the above reports, in a comparison between the use of hCG and r-hLH in women having at least 2 previous failed attempts at pregnancy, the number of follicles and oocytes and implantation and pregnancy rates were higher in the women receiving hCG [76].

A review of the literature, performed in 2012, on the use of exogenous LH in ART concluded that there was insufficient evidence for the general use of exogenous LH in GnRH antagonist cycles or the benefit of LH and hCG protocols. However, the authors suggested that poor responders and women over 35 years old may benefit from the administration of exogenous r-hLH [77]. The benefits of the addition of r-hLH in such specific sub-populations was confirmed in the latest and largest meta-analysis of 40 randomised, controlled trials of 6443 patients treated with r-hLH/r-hFSH vs FSH only, in women aged 18–45 years or older. The study showed significantly more oocytes were retrieved in poor responders treated with r-hFSH plus r-hLH vs r-hFSH alone (n = 1077; weighted mean difference +0.75 oocytes; 95% confidence interval [CI] 0.14–1.36). A significantly higher clinical pregnancy rate was observed with r-hLH/r-hFSH vs r-hFSH alone in the overall population (risk ratio [RR] 1.09; 95% CI 1.01–1.18). This difference was greater in poor responders (n = 1779; RR 1.30; 95% CI 1.01–1.67; intention-to-treat) [78].

A randomised trial of 96 patients undergoing IVF-ICSI showed that levels of amphiregulin — which have been linked to oocyte development and competence — were closer to those seen physiologically following the initiation of an endogenous LH surge by a GnRH agonist than with hCG [79]. This study also showed that significantly more metaphase II oocytes (+14%) and transferable embryos (+11%) were obtained with the GnRH agonist than with hCG. Moreover, a Cochrane review conducted in 2011 examined all randomised controlled trials (42 trials, 9606 couples) of rFSH vs urinary derived gonadotropins [80]. This review concluded that the differences in effectiveness and safety between the therapies were small.

A more recent matched pair study [81] of women receiving either r-hLH/r-hFSH or hMG during COS showed that pregnancy rates per cycle (p = 0.006; p = 0.022) and per embryo transfer (p = 0.025; p = 0.008), and implantation rate per embryo transferred (p <0.001; p <0.001) were significantly higher in the group treated with r-hLH/r-hFSH. Fábregues et al. also showed that the oocyte yield and the number of fertilised oocytes was higher in women treated with r-hFSH/r-hLH during COS compared with those treated with HP-hMG [82]. However, in that study, implantation and pregnancy rates were similar between groups. Finally, more oocytes were retrieved from women aged 18–35 years treated with r-hLH/r-hFSH compared with hMG in a recent prospective study [47].

The effect of LH supplementation may be more apparent in poor ovarian responders [83]. In a prospective study of mature oocytes and live birth rates in women treated with r-hLH/r-hFSH, HP-hMG or FSH, the number of mature oocytes and live birth rates were higher with LH-FSH than hMG and FSH in poor ovarian responders [83]. Further, a meta-analysis of seven randomised controlled trials of r-hLH/r-hFSH vs FSH only, in women aged 35 years or older, showed higher clinical pregnancy rates in those treated with r-hLH/r-hFSH [84]. While there was criticism of the detailed reporting of the meta-analysis, the overall conclusions drawn from these analyses were not materially altered [85]. These findings may help to explain the difference in outcomes of previous studies comparing the efficacy of r-hLH/r-hFSH and HP-hMG.

### Cost effectiveness of r-hLH compared with hCG

There are few current data to establish the cost effectiveness of r-hLH compared with hCG. The acquisition cost of r-hLH per treatment is certainly higher than that of HP-hMG. However, a recent cost-effectiveness study performed in Italy [86], found that due to higher pregnancy rates with –hFSH/r-hLH, the cost per pregnancy was higher for HP-hMG (€5,439.80) compared with r-hLH (€3,990.00). In support of this, a study by Carone et al. reported higher pregnancy rates in women receiving r-hFSH/r-hLH vs HP-hMG (58% vs 22% pregnancies in the first cycle respectively) [87].

## Conclusions

In this review we have described the differences in the structure and function of the gonadotropins LH and hCG, and have discussed how these differences may impact on the clinical outcomes of the use of recombinant or urinary therapies for ART.

In the natural cycle, the role of LH is to support normal follicular development over the course of approximately 14 days. In contrast, the natural physiological function of hCG is to support implantation and pregnancy over the course of approximately 9 months. However, when urinary ART therapies are used, hCG is added to urinary hMG to replace LH lost during the purification process and to standardise the therapy. While hCG has LH-like activity, it differs compared with LH in its potency and duration of action. The pharmacological dose of hCG in hMG may lead to excessive LH-like activity causing premature luteinisation, reduced fertilisation rates, and down regulation of the LH/hCG receptor expression in the follicular compartment.

r-hLH contains 99% pure LH, while the most advanced hMG available contains a mixture of FSH, LH and hCG with ~30% impurities, including significant biologically active contaminants such as growth factors, binding proteins and, importantly, prion proteins. Furthermore, the presence of other cytokines and molecules with biological reactivity may adversely affect successful pregnancy.

LH and hCG directly increase androgen and progesterone production and, thus, indirectly the production of oestrogens. However, intrafollicular progesterone is a terminal product that cannot be converted to oestradiol by LH or hCG due to lack of 3βHSD and P450-17α.

The lack of objective biomarkers of embryo viability makes it impossible to define a clear relationship between embryo quality and LH/hCG. However, the clinical evidence suggests that r-hLH may provide a more physiological support of follicle development in those categories of patients who require it. According to current thinking, patient subpopulations that may benefit from real LH supplementation include patients with hypogonadotrophic hypogonadism, patients who have profound LH suppression in a long GnRH agonist protocol, patients with a suboptimal response to FSH alone (9–25% of patients) and some patients older than 35 years.

Overall, while there is some clinical evidence to demonstrate differences between real LH and hCG for ovarian stimulation in ART, there is still a need for further randomised, controlled trials to provide clarification of the advantages of using one type of gonadotropin over another in different patient subsets.

## Abbreviations

3βHSD: 3-β-hydroxysteroid dehydrogenase
ART: Assisted reproductive technology
CAMP: Cyclic adenosine monophosphate
CJD: Creutzfeldt-Jacob disease
CI: Confidence interval
COS: Controlled ovarian stimulation
EGF: Epidermal growth factor
FSH: Follicle-stimulating hormone
GnRH: Gonadotrphin-releasing hormone
HCG: Human chorionic gonadotropin
ICSI: Intra-cytoplasmic sperm injection
IU: International units
HMG: Human menopausal gonadotropin
ICOS: Individualised controlled ovarian stimulation
IGF: Insulin-like growth factor
IV: Intravenous
IVF: *In* vitro fertilisation
LH: Luteinising hormone
RR: Risk ratio
SC: Subcutaneous
TSE: Transmissible spongiform encephalopathies
VEGF: Vascular endothelial growth factor.
